# Supplementary material for: Nonequilibrium photocarrier and phonon dynamics from first principles: a unified treatment of carrier-carrier, carrier-phonon, and phonon-phonon scattering
Source: NPJ Comput Mater. 2026 May 17;12(1):260. doi: 10.1038/s41524-026-02104-y (PMC13437248; doi:10.1038/s41524-026-02104-y)
Supplement: Supplementary file 1 [file 41524_2026_2104_MOESM1_ESM.pdf]

Supplementary Information: Nonequilibrium Photocarrier and Phonon Dynamics from First Principles: a Unified Treatment of Carrier-Carrier, Carrier-Phonon, and Phonon-Phonon Scattering

Stefano Mocatti, Giovanni Marini, Giulio Volpato, Pierluigi Cudazzo, Matteo Calandra

Department of Physics, University of Trento, Via Sommarive 14, Povo, 38123, Trento, Italy.

*Corresponding author(s). E-mail(s): stefano.mocatti@unitn.it; m.calandrabuonaura@unitn.it;

1 Second quantization operators

In the main text, we introduced the second-quantized creation and annihilation operators for electrons in the Kohn-Sham orbital $\psi_{\mathbf{k}n}(\mathbf{r},\sigma)$, denoted by $\hat{c}^{\dagger}_{\mathbf{k}n}$ and $\hat{c}_{\mathbf{k}n}$, respectively. These fermionic operators satisfy the canonical anticommutation relations:

$$\begin{aligned} \left\{\hat{c}_{\mathbf{k}n}, \hat{c}^{\dagger}_{\mathbf{k}'m}\right\} &= \delta_{\mathbf{k}\mathbf{k}'}\delta_{nm}, \\ \left\{\hat{c}^{\dagger}_{\mathbf{k}n}, \hat{c}^{\dagger}_{\mathbf{k}'m}\right\} &= \left\{\hat{c}_{\mathbf{k}n}, \hat{c}_{\mathbf{k}'m}\right\} = 0. \end{aligned} \tag{S1}$$

We similarly introduced the atomic displacement and momentum operators $\hat{Q}_{I\alpha}$ and $\hat{P}_{I\alpha}$. Upon applying a Fourier transformation, we obtained the bosonic creation and annihilation operators $\hat{a}^{\dagger}_{\mathbf{q}\nu}$ and $\hat{a}_{\mathbf{q}\nu}$, which respectively create and annihilate a vibrational quantum with momentum $\mathbf{q}$ and branch index ν. These operators obey the canonical commutation relations:

$$\begin{aligned} \left[\hat{a}_{\mathbf{q}\nu}, \hat{a}^{\dagger}_{\mathbf{q}'\nu'}\right] &= \delta_{\mathbf{q}\mathbf{q}'}\delta_{\nu\nu'}, \\ \left[\hat{a}^{\dagger}_{\mathbf{q}\nu}, \hat{a}^{\dagger}_{\mathbf{q}'\nu'}\right] &= \left[\hat{a}_{\mathbf{q}\nu}, \hat{a}_{\mathbf{q}'\nu'}\right] = 0. \end{aligned} \tag{S2}$$

From these, the commutation relations for the displacement and momentum operators in reciprocal space can be easily derived. These read

$$\begin{aligned} [Q_{\mathbf{q}\nu}, Q_{\mathbf{q}'\nu'}] &= [P_{\mathbf{q}\nu}, P_{\mathbf{q}'\nu'}] = 0, \\ [Q_{\mathbf{q}\nu}, P_{\mathbf{q}'\nu'}] &= i\delta_{\mathbf{q}'-\mathbf{q}}\delta_{\nu\nu'}. \end{aligned} \tag{S3}$$

2 Kadanoff-Baym equations

We briefly introduce the electronic Kadanoff-Baym equations (KBE). The starting point is the electronic single-particle Green's function:

$$G_{\mathbf{k}nm}(z,z') = -i\left\langle T_c\left[\hat{c}_{\mathbf{k}n}(z)\hat{c}^{\dagger}_{\mathbf{k}m}(z')\right]\right\rangle, \tag{S4}$$

where the creation and annihilation operators are in the Heisenberg representation and z and z' are complex times that lie on the Schwinger-Keldysh contour [1, 2]. Here, $\langle\,\rangle$ denotes a thermal average over the system's initial state, and T_c is the time-ordering operator along the contour.

We can define different real-time Green's function components: the lesser and greater parts

$$G^>_{\mathbf{k}nm}(t,t') = -i\langle \hat{c}_{\mathbf{k}m}(t)\hat{c}^\dagger_{\mathbf{k}n}(t')\rangle, \tag{S5}$$

$$G^<_{\mathbf{k}nm}(t,t') = \ i\langle \hat{c}^\dagger_{\mathbf{k}n}(t)\hat{c}_{\mathbf{k}m}(t')\rangle, \tag{S6}$$

and the retarded and advanced components

$$G^R_{\mathbf{k}nm}(t,t') = \theta(t-t')\left[G^>_{\mathbf{k}nm}(t,t') - G^<_{\mathbf{k}nm}(t,t')\right], \tag{S7}$$

$$G^A_{\mathbf{k}nm}(t,t') = \left[G^R_{\mathbf{k}nm}(t',t)\right]^\dagger. \tag{S8}$$

The self-energy $\Sigma_{\mathbf{k}nm}(z,z')$ is defined as the irreducible part of the Green's function [3] and defines the following Dyson equation:

$$G_{\mathbf{k}}(z,z') = \left[G^0_{\mathbf{k}} + G^0_{\mathbf{k}}\cdot\Sigma_{\mathbf{k}}\cdot G_{\mathbf{k}}\right](z,z'), \tag{S9}$$

where we used matrix notation for the band indices, and the "$\cdot$" indicates implicit time convolutions along the contour. The non-interacting Green's function $G^0_{\mathbf{k}}$ is obtained from Eq. (S4) by evolving the operators with the non-interacting time-dependent Hamiltonian $h_{\mathbf{k}}(t)$. This Hamiltonian may include an explicit time-dependent part due to external driving.

The greater and lesser components of the self-energy can be extracted from the general structure of any function defined on the contour:

$$F(z,z') = \theta(z,z')F^>(z,z') + \theta(z',z)F^<(z,z') + F^\delta(t)\delta(z,z'), \tag{S10}$$

where $\theta(z,z')$ and $\delta(z,z')$ are Heaviside and Dirac delta functions along the contour, and $F^\delta(z)$ is a time-local term accounting for a possible singular part. The retarded and advanced components are then given by

$$F^{R/A}(t,t') = \pm\theta(\pm t\mp t')\left[F^<(t,t') - F^>(t,t')\right] + F^\delta(t)\delta(t-t'). \tag{S11}$$

In the presence of an explicitly time-dependent perturbation, the EOMs for the contour single-particle Green's function coincide with the electronic Kadanoff-Baym equations [2–6], namely:

$$\Big[i\partial_t - h_{\mathbf{k}}(t)\Big]G^\lessgtr_{\mathbf{k}}(t,t') = \Big[\Sigma^R_{\mathbf{k}}\cdot G^\lessgtr_{\mathbf{k}} + \Sigma^\lessgtr_{\mathbf{k}}\cdot G^A_{\mathbf{k}}\Big](t,t'), \tag{S12}$$

Following the same conventions as in Eq. (S9), we use matrix notation for the band indices and denote time convolutions along the contour with "$\cdot$". The electronic occupations and polarizations are obtained from $G^<_{\mathbf{k}}(t,t')$ by evaluating it at equal times, i.e., by setting $t'=t$. We thus define

$$p_{\mathbf{k}}(t) = iG^<_{\mathbf{k}}(t,t), \tag{S13}$$

where the diagonal elements $p_{\mathbf{k}nn}(t)$ correspond to the occupations $f_{\mathbf{k}n}(t)$, and the off-diagonal elements $p_{\mathbf{k}nm}(t)$ $(n\neq m)$ are the polarizations.

The equation of motion for $p_{\mathbf{k}}(t)$ can be derived from Eq. (S12) by considering the equation for $G^<$, subtracting its Hermitian adjoint, and then setting $t'=t$. This yields

$$\frac{dp_{\mathbf{k}}(t)}{dt} + i[h^{qp}_{\mathbf{k}}(t), p_{\mathbf{k}}(t)] = \mathcal{I}_{\mathbf{k}}(t) + \mathcal{I}^\dagger_{\mathbf{k}}(t), \tag{S14}$$

where we defined the quasiparticle Hamiltonian $h^{qp}_{\mathbf{k}}(t)$ as

$$h^{qp}_{\mathbf{k}}(t) = h_{\mathbf{k}}(t) + \Sigma^\delta_{\mathbf{k}}(t), \tag{S15}$$

with $\Sigma^{\delta}_{\mathbf{k}}(t)$ being the singular (time-local) Hermitian part of the self-energy (see Eq. (S10)), and the collision integral

$$\mathcal{I}(t) = \int_{-\infty}^{t} d\bar{t} \left[\Sigma^{>}(t,\bar{t})G^{<}(\bar{t},t) - \Sigma^{<}(t,\bar{t})G^{>}(\bar{t},t)\right]. \tag{S16}$$

For the exact self-energy, the singular part corresponds to the Hartree-Fock self-energy. However, depending on the chosen approximation, it may also include additional time-local contributions. In fact, the self-energy can be written exactly as

$$\Sigma_{\mathbf{k}}(t,t') = \delta(t-t')\Sigma^{\mathrm{add}}_{\mathbf{k}}(t) + \Delta\Sigma_{\mathbf{k}}(t,t'), \tag{S17}$$

where $\Delta\Sigma_{\mathbf{k}}(t,t') = \Sigma_{\mathbf{k}}(t,t') - \delta(t-t')\Sigma^{\mathrm{add}}_{\mathbf{k}}(t)$. The singular part $\Sigma^{\mathrm{app}}_{\mathbf{k}}(t)$ includes additional singular contributions to the self-energy other than Hartree-Fock ones. This partition preserves the greater/lesser parts of the self-energy, hence

$$\Delta\Sigma^{\lessgtr}_{\mathbf{k}}(t,t') = \Sigma^{\lessgtr}_{\mathbf{k}}(t,t'). \tag{S18}$$

Therefore, we can include in the singular part of the self-energy additional static Hermitian contributions.

The role of the local self-energy in Eq. (S15) is to renormalize the single-particle Hamiltonian and, in some approximations, to yield a diagonal quasiparticle Hamiltonian.

For example, in the Hartree plus COHSEX and FM approximation for the self-energy (see Eqs. (S22)-(S29)), and in the absence of an external drive, we obtain a diagonal quasiparticle Hamiltonian within the no mixing mode approximation in the KS basis:

$$h^{qp}_{\mathbf{k}nm}(0) = h_{\mathbf{k}nm} + \Sigma^{\delta}_{\mathbf{k}nm}(0) = \delta_{nm}\varepsilon^{0}_{\mathbf{k}n}, \tag{S19}$$

where we have explicitly written the band indices and denoted the equilibrium quasiparticle energies by $\varepsilon^{0}_{\mathbf{k}n}$.

At finite times, both diagonal and off-diagonal renormalizations are governed by

$$\Delta\Sigma^{\delta}_{\mathbf{k}}(t) = \Sigma^{\delta}_{\mathbf{k}}(t) - \Sigma^{\delta}_{\mathbf{k}}(0).$$

In particular, in the presence of an external perturbation, this leads to a renormalization of the quasiparticle eigenvalues and Rabi frequencies.

The specific form of the collision integral in Eq. (S16) depends on the chosen self-energy approximation. However, Eq. (S14) is not closed, as the collision integral depends explicitly on the two-time Green's functions $G^{\lessgtr}(t,t')$. To address this issue, we adopt the Generalized Kadanoff-Baym Ansatz (GKBA) [7–10], whereby the lesser and greater Green's functions are approximated as

$$G^{\lessgtr}_{\mathbf{k}}(t,t') \simeq -G^{R}_{\mathbf{k}}(t,t')p_{\mathbf{k}}(t') + p_{\mathbf{k}}(t)G^{A}_{\mathbf{k}}(t,t'), \tag{S20}$$

which becomes exact in the non-interacting limit. The retarded Green's function is evaluated within the quasiparticle approximation:

$$G^{R}_{\mathbf{k}}(t,t') = -i\theta(t-t')\exp\left[-i\int_{t'}^{t} h^{qp}_{\mathbf{k}}(\bar{t})\,d\bar{t}\right], \tag{S21}$$

where, as a further approximation, the time dependence of $h^{qp}_{\mathbf{k}}(t)$ may be neglected. Assuming this Ansatz for the retarded (and advanced) propagator corresponds to disregarding part of the correlation self-energy's effect. Such an approximation is physically justified when quasiparticles are well-defined between collisions, i.e., when the average time between collisions is much shorter than the quasiparticle decay time.

By combining Eqs. (S14), (S16), (S20), and (S21), we obtain a closed set of equations for the electronic occupations and polarizations. Nonetheless, the time-nonlocal structure of the integrand in Eq. (S16) requires access to the full time history of the system and necessitates explicit integration at each time step. This significantly hinders computational efficiency, as the cost scales quadratically with the simulation time [11].

A significant simplification in this context is provided by the Markov approximation, which allows one to reformulate Eq. (S16) in a time-local form by eliminating the non-locality of the integrand. Details on the derivation of the collision integrals can be found in Refs. [10, 12].

3 Self-energy expressions

The time evolution of the electronic eigenvalues and Rabi frequencies, as well as the phonon frequencies, are governed by the time-local electron and phonon self-energies. Below, we provide explicit expressions for each self-energy contribution within the approximation scheme outlined in the main text.

The renormalization of electronic eigenvalues is described by $\Delta\Sigma_{\mathbf{k}n}(t)$ within the quasiparticle approximation. This time-dependent correction consists of four terms: Hartree (H), static GW, Fan-Migdal (FM), and coherent atomic motion (AM):

$$\Delta\Sigma_{\mathbf{k}n}(t) = \Delta\Sigma^{\mathrm{H}}_{\mathbf{k}n}(t) + \Delta\Sigma^{\mathrm{AM}}_{\mathbf{k}n}(t) + \Delta\Sigma^{\mathrm{GW}}_{\mathbf{k}n}(t) + \Delta\Sigma^{\mathrm{FM}}_{\mathbf{k}n}(t). \tag{S22}$$

The Hartree self-energy captures the variation of the Hartree potential due to changes in electron density. It reads

$$\Delta\Sigma^{\mathrm{H}}_{\mathbf{k}n}(t) = \frac{1}{N}\sum_{\mathbf{k}',n'} \widetilde{v}^{nn'nn'}_{\mathbf{k}\mathbf{k}'\mathbf{k}\mathbf{k}'}(\mathbf{0})\Delta f_{\mathbf{k}'n'}(t), \tag{S23}$$

where $\widetilde{v}$ denotes the non-homogeneous part of the Coulomb interaction in reciprocal space:

$$\widetilde{v}^{nn'nn'}_{\mathbf{k}\mathbf{k}'\mathbf{k}\mathbf{k}'}(\mathbf{q}) = \sum_{\mathbf{G}\neq\mathbf{0}} \left(\rho^{\mathbf{G}}_{nn}(\mathbf{k},\mathbf{0})\right)^* v_{\mathbf{G}}(\mathbf{q})\rho^{\mathbf{G}}_{n'n'}(\mathbf{k}',\mathbf{0}), \tag{S24}$$

with

$$v_{\mathbf{G}}(\mathbf{q}) = \frac{8\pi}{|\mathbf{q}+\mathbf{G}|^2}, \tag{S25}$$

the Fourier transform of the Coulomb potential and $\mathbf{G}$ a reciprocal lattice vector. As noted in the main text, this term reflects the effect of non-uniform density fluctuations, i.e., a local-field correction. As a further note, we highlight that the homogeneous component ($\mathbf{G} = 0$) is not considered here due to charge neutrality.

The atomic motion self-energy arises from the electron-phonon tadpole diagram [13], involving two bare electron-phonon vertices. Upon solving the Dyson equation self-consistently, one vertex becomes dressed [13], and the diagram reduces to a contribution proportional to the average atomic displacement [2, 14]:

$$\Delta\Sigma^{\mathrm{AM}}_{\mathbf{k}n}(t) = \sum_{\nu} g^{\nu}_{nn}(\mathbf{k},\mathbf{0})Q_{\nu}(t), \tag{S26}$$

where $Q_\nu(t)$ is the coherent phonon amplitude. This term is consistent with the DFPT expression for the variation of KS eigenvalues under atomic displacement.

The static GW term describes the energy shift from excited carriers within the COHSEX approximation [15–18]. It can be split into screened exchange (SEX) and Coulomb hole (COH) components:

$$\Delta\Sigma^{\mathrm{GW}}_{\mathbf{k}n}(t) = \Delta\Sigma^{\mathrm{SEX}}_{\mathbf{k}n}(t) + \Delta\Sigma^{\mathrm{COH}}_{\mathbf{k}n}(t). \tag{S27}$$

Both terms are computed as the difference between their nonequilibrium and equilibrium values:

$$\Delta\Sigma^{\mathrm{GW}}_{\mathbf{k}n}(t) = -\frac{1}{N}\sum_{\mathbf{k}',m}\left[W^{nmmn}_{\mathbf{k}\mathbf{k}'\mathbf{k}'\mathbf{k}}(t)\,\Delta f_{\mathbf{k}'m}(t) - \left(\frac{1}{2} - f_{\mathbf{k}'m}(0)\right)\left(W^{nmmn}_{\mathbf{k}\mathbf{k}'\mathbf{k}'\mathbf{k}}(t) - W^{nmmn}_{\mathbf{k}\mathbf{k}'\mathbf{k}'\mathbf{k}}(0)\right)\right]. \tag{S28}$$

The first term contributes only for states with time-dependent occupations, while the second accounts for screening changes due to all states.

The time-dependent Fan-Migdal self-energy is computed by subtracting the equilibrium real part and adding the nonequilibrium correction [19, 20]:

$$\Sigma^{\mathrm{FM}}_{\mathbf{k}n}(t) = \frac{1}{N}\sum_{\mathbf{q},\nu,m}|g^{\nu}_{mn}(\mathbf{k},\mathbf{q})|^2\mathscr{P}\left[\frac{n_{\mathbf{q}\nu}(t)+f_{\mathbf{k}+\mathbf{q}m}(t)}{\varepsilon^{\mathrm{KS}}_{\mathbf{k}n}-\varepsilon^{\mathrm{KS}}_{\mathbf{k}+\mathbf{q}m}+\omega^0_{\mathbf{q}\nu}} + \frac{n_{\mathbf{q}\nu}(t)+1-f_{\mathbf{k}+\mathbf{q}m}(t)}{\varepsilon^{\mathrm{KS}}_{\mathbf{k}n}-\varepsilon^{\mathrm{KS}}_{\mathbf{k}+\mathbf{q}m}-\omega^0_{\mathbf{q}\nu}}\right], \tag{S29}$$

where $\mathscr{P}$ denotes the regularized Cauchy principal value and $g^{\nu}_{nm}(\mathbf{k},\mathbf{q})$ is the electron-phonon matrix defined in the main text. The self-energy variation is given by $\Delta\Sigma(t)=\Sigma(t)-\Sigma(0)$.

The off-diagonal self-energy modifies the Rabi frequencies. Including the SEX term while neglecting collisions yields the real-time Bethe-Salpeter equation in linear response [21]. Here, we neglect all terms other than the SEX contribution, obtaining:

$$\Delta\Sigma_{\mathbf{k}nm}(t)=-\frac{1}{N}\sum_{\mathbf{k}',n',m'}W^{\mathbf{k}\mathbf{k}'\mathbf{k}'\mathbf{k}}_{nn'm'm}(t)\,p_{\mathbf{k}'m'n'}(t). \tag{S30}$$

Phonon frequency renormalization is controlled by the phonon self-energy $\Delta\Pi_{\mathbf{q}\nu}(t)$. As discussed in the main text, we consider contributions from electron-hole (EH) and anharmonic phonon bubble (PB) diagrams:

$$\Delta\Pi_{\mathbf{q}\nu}(t)=\Delta\Pi^{\mathrm{EH}}_{\mathbf{q}\nu}(t)+\Pi^{\mathrm{PB}}_{\mathbf{q}\nu}(t). \tag{S31}$$

In the adiabatic approximation, the EH contribution takes the form [22–24]:

$$\Pi^{\mathrm{EH}}_{\mathbf{q}\nu\nu'}(t)=\frac{1}{N}\sum_{\mathbf{k},n,m}\left(g^{\nu}_{mn}(\mathbf{k},\mathbf{q})\right)^{*}g^{\nu'}_{mn}(\mathbf{k},\mathbf{q})\mathscr{P}\left[\frac{f_{\mathbf{k}n}(t)-f_{\mathbf{k}+\mathbf{q}m}(t)}{\varepsilon^{\mathrm{KS}}_{\mathbf{k}n}-\varepsilon^{\mathrm{KS}}_{\mathbf{k}+\mathbf{q}m}}\right]. \tag{S32}$$

The corresponding variation is $\Delta\Pi(t)=\Pi(t)-\Pi(0)$ and we consider diagonal matrix elements only.

The phonon bubble contribution accounts for anharmonic phonon-phonon interactions [25–27]. Its real part reads:

$$\begin{aligned}\Pi^{\mathrm{PB}}_{\mathbf{q}\nu}(t)=\frac{1}{N}\sum_{\substack{\mathbf{q}_1,\mathbf{q}_2\\ \nu_1,\nu_2}}|\Phi^{(3)}_{\nu\nu_1\nu_2}(\mathbf{q},\mathbf{q}_1,\mathbf{q}_2)|^2\Bigg[&\frac{\left(1+n_{\mathbf{q}_1\nu_1}(t)+n_{\mathbf{q}_2\nu_2}(t)\right)\left(\omega^0_{\mathbf{q}_2\nu_2}+\omega^0_{\mathbf{q}_1\nu_1}\right)}{\left(\omega^0_{\mathbf{q}_1\nu_1}+\omega^0_{\mathbf{q}_2\nu_2}\right)^2-\left(\omega^0_{\mathbf{q}\nu}\right)^2}-\\ &\frac{\left(n_{\mathbf{q}_1\nu_1}(t)-n_{\mathbf{q}_2\nu_2}(t)\right)\left(\omega^0_{\mathbf{q}_2\nu_2}-\omega^0_{\mathbf{q}_1\nu_1}\right)}{\left(\omega^0_{\mathbf{q}_1\nu_1}-\omega^0_{\mathbf{q}_2\nu_2}\right)^2-\left(\omega^0_{\mathbf{q}\nu}\right)^2}\Bigg],\end{aligned} \tag{S33}$$

where $\Phi^{(3)}_{\nu\nu_1\nu_2}(\mathbf{q},\mathbf{q}_1,\mathbf{q}_2)$ is the third-order anharmonic coupling matrix.

4 Time-dependent RPA screening

The statically screened Coulomb interaction is time-dependent through the static RPA dielectric function, which varies due to changes in electronic occupations. Within RPA, the static dielectric function is determined by the irreducible density-density response function:

$$\chi^0_{\mathbf{G}\mathbf{G}'}(\mathbf{q},t)=\frac{1}{N}\sum_{\mathbf{k},n,m}\frac{f_{\mathbf{k}n}(t)-f_{\mathbf{k}+\mathbf{q}m}(t)}{\varepsilon^0_{\mathbf{k}n}-\varepsilon^0_{\mathbf{k}+\mathbf{q}m}+i\eta}\left(\rho^{\mathbf{G}}_{nm}(\mathbf{k},\mathbf{q})\right)^{*}\rho^{\mathbf{G}'}_{nm}(\mathbf{k},\mathbf{q}), \tag{S34}$$

where η is a positive infinitesimal and $\rho^{\mathbf{G}}_{nm}(\mathbf{k},\mathbf{q})$ is the oscillator strength.

The RPA dielectric matrix then reads:

$$\epsilon_{\mathbf{G}\mathbf{G}'}(\mathbf{q},t)=\delta_{\mathbf{G}\mathbf{G}'}-v_{\mathbf{G}}(\mathbf{q})\chi^0_{\mathbf{G}\mathbf{G}'}(\mathbf{q},t). \tag{S35}$$

We separate the response function into equilibrium (time-independent) and nonequilibrium (time-dependent) parts:

$$\chi^0_{\mathbf{G}\mathbf{G}'}(\mathbf{q},t)=\chi^0_{\mathbf{G}\mathbf{G}'}(\mathbf{q},0)+\Delta\chi^0_{\mathbf{G}\mathbf{G}'}(\mathbf{q},t), \tag{S36}$$

with $\Delta\chi^0_{\mathbf{G}\mathbf{G}'}(\mathbf{q},t)=\chi^0_{\mathbf{G}\mathbf{G}'}(\mathbf{q},t)-\chi^0_{\mathbf{G}\mathbf{G}'}(\mathbf{q},0)$. Accordingly, the dielectric matrix is partitioned as:

$$\epsilon_{\mathbf{G}\mathbf{G}'}(\mathbf{q},t)=\sum_{\mathbf{G}_1}\epsilon_{\mathbf{G}\mathbf{G}_1}(\mathbf{q},0)\Delta\epsilon_{\mathbf{G}_1\mathbf{G}'}(\mathbf{q},t), \tag{S37}$$

where the time-dependent correction is

$$\Delta\epsilon_{\mathbf{G}\mathbf{G}'}(\mathbf{q},t)=\delta_{\mathbf{G}\mathbf{G}'}-\sum_{\mathbf{G}_1}W_{\mathbf{G}\mathbf{G}_1}(\mathbf{q},0)\Delta\chi^0_{\mathbf{G}_1\mathbf{G}'}(\mathbf{q},t), \tag{S38}$$

and the equilibrium statically screened Coulomb interaction is defined as

$$W_{\mathbf{GG}'}(\mathbf{q},0) = \epsilon^{-1}_{\mathbf{GG}'}(\mathbf{q},0)v_{\mathbf{G}'}(\mathbf{q}). \tag{S39}$$

From Eq. (S37), the time-dependent screened Coulomb interaction becomes [28]:

$$W_{\mathbf{GG}'}(\mathbf{q},t) = \sum_{\mathbf{G}_1} \Delta\epsilon^{-1}_{\mathbf{GG}_1}(\mathbf{q},t)W_{\mathbf{G}_1\mathbf{G}'}(\mathbf{q},0). \tag{S40}$$

Thus, once the equilibrium screened interaction is known, its time evolution follows from the inverse of $\Delta\epsilon_{\mathbf{GG}'}(\mathbf{q},t)$ obtained via Eqs. (S34), (S36), and (S38).

5 Interpretation of rates through RTA

The electronic decay rates follow from the collision integrals via the relaxation-time approximation (RTA) and the quasi-equilibrium hypothesis. We assume that, at each time, the electron in the nth KS state with momentum $\mathbf{k}$ is close to equilibrium, and its deviation is captured by $\delta f_{\mathbf{k}n}$. This implies that the collision integral depends on $\delta f_{\mathbf{k}n}$ and vanishes when $\delta f_{\mathbf{k}n} = 0$.

Under these assumptions, we obtain

$$\mathcal{I}_{\mathbf{k}n}(t) = -2\delta f_{\mathbf{k}n}\Gamma_{\mathbf{k}n}(t), \tag{S41}$$

which defines the time-dependent RTA decay rate for the electronic state.

The same reasoning applies to the phonon scattering integrals, yielding the phonon RTA decay rates:

$$\mathcal{I}_{\mathbf{q}\nu}(t) = -2\delta n_{\mathbf{q}\nu}\Gamma_{\mathbf{q}\nu}(t), \tag{S42}$$

where $\delta n_{\mathbf{q}\nu}$ denotes the deviation from equilibrium of mode ν at momentum $\mathbf{q}$.

These RTA decay rates connect to the imaginary part of the equilibrium electron and phonon self-energies.

For electrons, the imaginary part of the self-energy at equilibrium arises from the static second-Born (SB) and Fan-Migdal (FM) contributions. The equilibrium diagonal static second Born self-energy is

$$\Sigma^{\mathrm{SB}}_{\mathbf{k}n}(\omega) = \frac{1}{N^2}\sum_{\substack{\mathbf{k}',\mathbf{q}\\ m,m',n'}} \left| W^{\mathbf{kk}'\mathbf{k}-\mathbf{qk}'+\mathbf{q}}_{nn'mm'} - W^{\mathbf{kk}'\mathbf{k}'+\mathbf{qk}-\mathbf{q}}_{nn'm'm}\right|^2 \frac{\left[f^0_{\mathbf{k}'+\mathbf{q}m'}(f^0_{\mathbf{k}-\mathbf{q}m} - f^0_{\mathbf{k}'n'}) + (1 - f^0_{\mathbf{k}n}f^0_{\mathbf{k}'n'})\right]}{\omega + \varepsilon^0_{\mathbf{k}'n'} - \varepsilon^0_{\mathbf{k}-\mathbf{q}m} - \varepsilon^0_{\mathbf{k}'+\mathbf{q}m'} + i\eta}, \tag{S43}$$

the Fan-Migdal self-energy reads [19, 20]

$$\Sigma^{\mathrm{FM}}_{\mathbf{k}n}(\omega) = \frac{1}{N}\sum_{\mathbf{q},\nu,m} |g^{\nu}_{mn}(\mathbf{k},\mathbf{q})|^2 \left[\frac{n^0_{\mathbf{q}\nu} + f^0_{\mathbf{k}+\mathbf{q}m}}{\omega - \varepsilon^0_{\mathbf{k}+\mathbf{q}m} + \omega^0_{\mathbf{q}\nu} + i\eta} + \frac{n^0_{\mathbf{q}\nu} + 1 - f^0_{\mathbf{k}+\mathbf{q}m}}{\omega - \varepsilon^0_{\mathbf{k}+\mathbf{q}m} - \omega^0_{\mathbf{q}\nu} + i\eta}\right], \tag{S44}$$

with η a positive infinitesimal. Evaluating the self-energies at the quasiparticle energy and taking the imaginary part gives

$$-\,\mathrm{Im}\left[\Sigma^{\mathrm{SB}}_{\mathbf{k}n}(\varepsilon^0_{\mathbf{k}n}) + \Sigma^{\mathrm{FM}}_{\mathbf{k}n}(\varepsilon^0_{\mathbf{k}n})\right] = \Gamma^{ee}_{\mathbf{k}n}(0) + \Gamma^{ep}_{\mathbf{k}n}(0). \tag{S45}$$

For phonons, the equilibrium self-energy includes the electron-hole (EH) and phonon-bubble (PB) diagrams. The electron-hole contribution reads [20, 22, 29]

$$\Pi^{\mathrm{EH}}_{\mathbf{q}\nu}(\omega) = \frac{1}{N}\sum_{\mathbf{k},n,m} |g^{\nu}_{mn}(\mathbf{k},\mathbf{q})|^2 \frac{f^0_{\mathbf{k}+\mathbf{q}m} - f^0_{\mathbf{k}n}}{\varepsilon^0_{\mathbf{k}+\mathbf{q}m} - \varepsilon^0_{\mathbf{k}n} - \omega - i\eta}, \tag{S46}$$

and the phonon bubble term is [25–27]

$$\Pi^{\mathrm{PB}}_{\mathbf{q}\nu}(\omega) = -\frac{1}{2N}\sum_{\substack{\mathbf{q}',\mathbf{q}''\\ \nu',\nu''}} |\Phi^{(3)}_{\nu\nu'\nu''}(-\mathbf{q},\mathbf{q}',\mathbf{q}'')|^2 \left[\frac{(\omega^0_{\mathbf{q}'\nu'} + \omega^0_{\mathbf{q}''\nu''})(1 + n^0_{\mathbf{q}'\nu} + n^0_{\mathbf{q}''\nu''})}{(\omega^0_{\mathbf{q}'\nu'} + \omega^0_{\mathbf{q}''\nu''})^2 - (\omega + i\eta)^2} + \frac{(\omega^0_{\mathbf{q}''\nu''} - \omega^0_{\mathbf{q}'\nu'})(n^0_{\mathbf{q}'\nu} - n^0_{\mathbf{q}''\nu''})}{(\omega^0_{\mathbf{q}'\nu'} - \omega^0_{\mathbf{q}''\nu''})^2 - (\omega + i\eta)^2}\right]. \tag{S47}$$

The equilibrium RTA phonon decay rate is then

$$-\,\mathrm{Im}\left[\Pi^{\mathrm{EH}}_{\mathbf{q}\nu}(\omega^0_{\mathbf{q}\nu})+\Pi^{\mathrm{PB}}_{\mathbf{q}\nu}(\omega^0_{\mathbf{q}\nu})\right]=\Gamma^{pe}_{\mathbf{q}\nu}(0)+\Gamma^{pp}_{\mathbf{q}\nu}(0). \tag{S48}$$

Thus, the equilibrium RTA decay rates correspond to the quasiparticle linewidths for both electrons and phonons. While Eqs. (S45) and (S48) hold at equilibrium, time dependence can be introduced through the nonequilibrium evolution of occupations, eigenvalues, and frequencies. This corresponds to choosing a time-dependent local form for the self-energies, providing a natural framework to evaluate quasiparticle lifetimes out of equilibrium.

6 Non-equilibrium ionic forces from Janak's theorem

The goal is to derive the light-induced forces from the DFT total energy due to a nonequilibrium electronic population [30]. To this end, we employ Janak's theorem [31] to express the energy variation due to small changes in electronic occupations. The total energy difference for fixed ionic positions is

$$\Delta E=\frac{1}{N}\sum_{\mathbf{k},n}\Delta f_{\mathbf{k}n}\varepsilon^{\mathrm{KS}}_{\mathbf{k}n}, \tag{S49}$$

where $\Delta f_{\mathbf{k}n}=f_{\mathbf{k}n}-f^0_{\mathbf{k}n}$ denotes the deviation from equilibrium. The force on atom a in cell I follows from the spatial derivative:

$$\begin{aligned}F_{Ia\alpha}&=-\frac{\partial\Delta E}{\partial Q_{Ia\alpha}}=-\frac{1}{N}\sum_{\mathbf{k},n}\Delta f_{\mathbf{k}n}\frac{\partial\varepsilon^{\mathrm{KS}}_{\mathbf{k}n}}{\partial Q_{Ia\alpha}}\\&=-\frac{1}{N}\sum_{\mathbf{k},n}\Delta f_{\mathbf{k}n}\left\langle\psi_{\mathbf{k}n}|d_{Ia\alpha}(\hat{\mathbf{r}})|\psi_{\mathbf{k}n}\right\rangle,\end{aligned} \tag{S50}$$

in agreement with the result reported in the main text.

7 Anharmonic effects on coherent phonons

We start from a model phonon Hamiltonian composed of the equilibrium BO harmonic part and the third-order anharmonic contribution:

$$\hat{H}=\hat{H}_{BO}+\hat{H}^{(3)}_{pp}, \tag{S51}$$

as defined in the main text. We neglect the fourth-order term, as its effect can be added on top of the results of the present section.

Our goal is to derive the equation of motion for the atomic displacement in reciprocal space. We focus on the zero-momentum component, assuming that crystal symmetry is preserved during the dynamics. Applying the Heisenberg equations of motion yields:

$$\frac{d^2Q_\nu(t)}{dt^2}=-\left[\omega^0_{\mathbf{0}\nu}\right]^2Q_\nu(t)-\sqrt{\frac{2(\omega^0_{\mathbf{0}\nu})^2}{N}}\sum_{\substack{\mathbf{q}\\\nu_1,\nu_2}}\Phi^{(3)}_{\nu\nu_1\nu_2}(0,\mathbf{q},-\mathbf{q})\langle Q_{\mathbf{q}\nu_1}Q_{-\mathbf{q}\nu_2}\rangle_t. \tag{S52}$$

Thus, the anharmonic contribution is governed by the equal-time displacement-displacement correlation function. In the absence of coherent atomic motion and neglecting all quadratic correlations except for the phonon occupations, we obtain:

$$\langle Q_{\mathbf{q}\nu}Q_{-\mathbf{q}\nu'}\rangle_t=\delta_{\nu\nu'}\frac{1+n_{\mathbf{q}\nu}(t)+n_{-\mathbf{q}\nu}(t)}{2}. \tag{S53}$$

Substituting this into Eq.(S52) yields the expression for the anharmonic force reported in the main text. In the following, we derive the contribution from coherent motion to the displacement-displacement correlation due to anharmonicity.

To this end, we consider the EOMs for the displacement-displacement correlation function:

$$\langle Q_{\mathbf{q}\nu}Q_{-\mathbf{q}\nu'}\rangle_t=\frac{1}{2}\left[\langle a_{\mathbf{q}\nu}a_{-\mathbf{q}\nu'}\rangle_t+\langle a^\dagger_{-\mathbf{q}\nu}a^\dagger_{\mathbf{q}\nu'}\rangle_t+\langle a_{\mathbf{q}\nu}a^\dagger_{\mathbf{q}\nu'}\rangle_t+\langle a^\dagger_{-\mathbf{q}\nu}a_{-\mathbf{q}\nu'}\rangle_t\right]. \tag{S54}$$

The EOMs for these correlations follow from the Heisenberg equations, leading to an infinite BBGKY hierarchy. We truncate this hierarchy using a mean-field approximation, assuming that coherent motion implies $\langle \hat{a}_{\mathbf{q}\nu} \rangle_t = \delta_{\mathbf{q0}} \langle \hat{a}_{\mathbf{0}\nu} \rangle_t$.

Within this approximation, third-order correlations are factorized into products of second- and first-order terms, and we retain only the phonon occupation among quadratic correlations:

$$\langle \hat{a}^\dagger_{\mathbf{q}\nu} \hat{a}^\dagger_{-\mathbf{q}\nu'} \rangle_t = \langle \hat{a}_{\mathbf{q}\nu} \hat{a}_{-\mathbf{q}\nu'} \rangle_t = 0, \tag{S55}$$

$$\langle \hat{a}^\dagger_{\mathbf{q}\nu} \hat{a}_{\mathbf{q}\nu'} \rangle_t = \langle \hat{a}_{\mathbf{q}\nu'} \hat{a}^\dagger_{\mathbf{q}\nu} \rangle_t - \delta_{\nu\nu'} = \delta_{\nu\nu'} n_{\mathbf{q}\nu}(t). \tag{S56}$$

This yields the following EOMs for the second-order correlations in Eq.(S54):

$$i\frac{d}{dt}\langle a^\dagger_{-\mathbf{q}\nu} a^\dagger_{\mathbf{q}\nu'} \rangle_t = -\left(\omega^0_{-\mathbf{q}\nu} + \omega^0_{\mathbf{q}\nu'}\right)\langle a^\dagger_{-\mathbf{q}\nu} a^\dagger_{\mathbf{q}\nu'} \rangle_t - \sqrt{\frac{2}{N}} \sum_{\nu''} \Phi^{(3)}_{\nu\nu'\nu''}(-\mathbf{q}, +\mathbf{q}, 0)\big[1 + n_{-\mathbf{q}\nu}(t) + n_{\mathbf{q}\nu'}(t)\big] Q_{\nu''}(t), \tag{S57}$$

$$i\frac{d}{dt}\langle a_{\mathbf{q}\nu} a^\dagger_{\mathbf{q}\nu'} \rangle_t = \left(\omega^0_{\mathbf{q}\nu} - \omega^0_{\mathbf{q}\nu'}\right)\langle a_{\mathbf{q}\nu} a^\dagger_{\mathbf{q}\nu'} \rangle_t + \sqrt{\frac{2}{N}} \sum_{\nu''} \Phi^{(3)}_{\nu\nu'\nu''}(\mathbf{q}, -\mathbf{q}, 0)\big[n_{\mathbf{q}\nu}(t) - n_{\mathbf{q}\nu'}(t)\big] Q_{\nu''}(t), \tag{S58}$$

while the remaining two correlations in Eq.(S54) follow by Hermitian conjugation or from the equal-time commutation relations, see Eq.(S2).

As a further simplification, we assume phonon occupations evolve slowly, so their time dependence can be neglected. The resulting EOMs for the quadratic correlators can be solved in Fourier space using Green's function techniques. Applying this to Eq.(S54), we find:

$$\sqrt{\frac{2}{N}} \sum_{\substack{\mathbf{q} \\ \nu,\nu'}} \Phi^{(3)}_{\bar{\nu}\nu\nu'}(0, \mathbf{q}, -\mathbf{q}) \langle Q_{\mathbf{q}\nu} Q_{-\mathbf{q}\nu'} \rangle_\omega = 2 Q_{\bar{\nu}}(\omega) \Pi^{\mathrm{PB}}_{\mathbf{0}\bar{\nu}}(\omega), \tag{S59}$$

where ω is the Fourier frequency and Π^{PB} is the phonon bubble anharmonic self-energy, see Eq.(S33). Inserting this into Eq.(S52), we find that the coherent phonon frequency is renormalized by the real part of the self-energy, while the imaginary part leads to damping.

Assuming a monochromatic perturbation at the unperturbed phonon frequency, we obtain:

$$\frac{d^2 Q_\nu(t)}{dt^2} = -\,\widetilde{\omega}^2_{\mathbf{0}\nu} Q_\nu(t) - 2\Gamma^{pp}_{\mathbf{0}\nu} \frac{dQ_\nu(t)}{dt} + F^{pp}_\nu, \tag{S60}$$

with

$$\widetilde{\omega}^2_{\mathbf{0}\nu} = \left[\omega^0_{\mathbf{0}\nu}\right]^2 + 2\omega^0_{\mathbf{0}\nu} \operatorname{Re}[\Pi^{\mathrm{PB}}_{\mathbf{0}\nu}(\omega^0_{\mathbf{0}\nu})], \tag{S61}$$

$$\Gamma^{pp}_{\mathbf{0}\nu} = -\operatorname{Im}[\Pi^{\mathrm{PB}}_{\mathbf{0}\nu}(\omega^0_{\mathbf{0}\nu})], \tag{S62}$$

$$F^{pp}_\nu = -\sqrt{\frac{(\omega^0_{\mathbf{0}\nu})^2}{2N}} \sum_{\mathbf{q},\bar{\nu}} \Phi^{(3)}_{\nu\bar{\nu}\bar{\nu}}(\mathbf{0}, \mathbf{q}, -\mathbf{q})\big(1 + n_{\mathbf{q}\bar{\nu}} + n_{-\mathbf{q}\bar{\nu}}\big).$$

Finally, we can restore the time dependence of phonon occupations, which was neglected in the derivation. This results in time-dependent frequencies, damping, and forces.

8 Explicit time-propagation

Within an explicit time-propagation scheme and given the first-order Cauchy problem:

$$\begin{cases} \mathbf{y}'(t) = \mathbf{f}(\mathbf{y}, t), \\ \mathbf{y}(t_0) = \mathbf{y}_0, \end{cases} \tag{S63}$$

c_i		
0		
$\frac{1}{2}$	$\frac{1}{2}$	
	0	1

RK2

c_i				
0				
$\frac{1}{2}$	$\frac{1}{2}$			
$\frac{1}{2}$	0	$\frac{1}{2}$		
1	0	0	1	
	$\frac{1}{6}$	$\frac{1}{3}$	$\frac{1}{3}$	$\frac{1}{6}$

RK4

c_i							
0							
$\frac{1}{5}$	$\frac{1}{5}$						
$\frac{3}{10}$	$\frac{3}{40}$	$\frac{9}{40}$					
$\frac{4}{5}$	$\frac{44}{45}$	$-\frac{56}{15}$	$\frac{32}{9}$				
$\frac{8}{9}$	$\frac{19372}{6561}$	$-\frac{25360}{2187}$	$\frac{64448}{6561}$	$-\frac{212}{729}$			
1	$\frac{9017}{3168}$	$-\frac{355}{33}$	$\frac{46732}{5247}$	$\frac{49}{176}$	$-\frac{5103}{18656}$		
1	$\frac{35}{384}$	0	$\frac{500}{1113}$	$\frac{125}{192}$	$-\frac{2187}{6784}$	$\frac{11}{84}$	
	$\frac{35}{384}$	0	$\frac{500}{1113}$	$\frac{125}{192}$	$-\frac{2187}{6784}$	$\frac{11}{84}$	0
	$\frac{5179}{57600}$	0	$\frac{7571}{16695}$	$\frac{393}{640}$	$-\frac{92097}{339200}$	$\frac{187}{2100}$	$\frac{1}{40}$

RKDP54

Table S1: Butcher tableaux for the RK2, RK4, and RKDP5(4) methods. The left column contains the c_i coefficients, the lower triangular matrix contains the a_{ij} coefficients, and the bottom row contains the b_i. The RKDP5(4) method has two sets of weights b_i, one for each order proposal.

the value of $\mathbf{y}$ at $t_{i+1} = t_i + \Delta t$, namely $\mathbf{y}_{i+1} = \mathbf{y}(t_{i+1})$, is obtained as:

$$\mathbf{y}_{i+1} = \mathbf{y}_i + \Delta t \sum_{j=1}^{n} b_j \mathbf{k}_j + O(\Delta t^M), \tag{S64}$$

where

$$\mathbf{k}_j = \mathbf{f}\left(\mathbf{y}_i + \Delta t \sum_{l<j} a_{jl}\mathbf{k}_l, t_i + c_j \Delta t\right). \tag{S65}$$

The coefficients b_j, a_{jl}, and c_j form the *Butcher tableau*, which uniquely determines the explicit RK algorithm. The integer n denotes the number of evaluations of the function $\mathbf{f}$ required for the evolution. The number M corresponds to the order of the error associated with a single timestep propagation (see Eq. (S64)).

We implemented second-order (RK2), fourth-order (RK4), and fifth/fourth-order Runge-Kutta-Dormand-Prince (RKDP54) [32] algorithms. The RKDP54 method belongs to the class of adaptive stepsize algorithms, where the timestep Δt_i is adjusted at every step of the dynamics to keep the error below a given bound. The error is estimated from the difference between two proposals for $\mathbf{y}_{i+1}$: the first of fourth order, $\mathbf{y}^{(4)}_{i+1}$, and the second of fifth order, $\mathbf{y}^{(5)}_{i+1}$. These two estimates are obtained from the same rates $\mathbf{k}_j$ but using different values of the coefficients b_j, see Eq.(S64). The error ϵ is calculated as:

$$\epsilon = \max_j \left| \left(\mathbf{y}^{(5)}_{i+1}\right)_j - \left(\mathbf{y}^{(4)}_{i+1}\right)_j \right|. \tag{S66}$$

A new timestep Δt_{i+1} is then proposed to keep the error below a given threshold ϵ_{tol}:

$$\Delta t_{i+1} = \min\left(\max\left(0.9\left(\frac{\epsilon_{\text{tol}}}{\epsilon}\right)^{1/5}, \frac{1}{4}\right), 4\right)\Delta t_i. \tag{S67}$$

If $\epsilon < \epsilon_{\text{tol}}$, we set $\mathbf{y}_{i+1} = \mathbf{y}^{(5)}_{i+1}$ and proceed to the next step. Otherwise, we recalculate fourth and fifth order estimates for $\mathbf{y}_{i+1}$ and calculate the error on the propagation with the new timestep. This is repeated until $\epsilon < \epsilon_{\text{tol}}$.

The Butcher tableau corresponding to RK2, RK4, and RKDP45 is reported in Tab.S1.

9 Ground-state model for the dielectric function without local-field effects

In the absence of local-field effects, the equilibrium screening entering the Coulomb interaction matrix, see Eq. (S39), is described through an effective two-dimensional dielectric model. Combined with the photoinduced contribution of Eq. (S38), this provides the real-time Coulomb matrix used in the simulations.

This choice is particularly suitable for suspended atomically thin semiconductors, where finite-thickness effects are essential for a realistic description of screening.

Specifically, following Ref. [33], we approximate the macroscopic component of the screened interaction as

$$W_{00}(\mathbf{q},0)=\frac{v_{00}^{2D}(q)}{\epsilon^{2D}(q)}, \tag{S68}$$

where $q=|\mathbf{q}|$, $v_{00}^{2D}(q)$ is the effective two-dimensional Coulomb interaction including finite-thickness corrections, and $\epsilon^{2D}(q)$ is the corresponding effective dielectric function. For a suspended 2D semiconductor, the latter is taken as

$$\epsilon^{2D}(q)=\frac{\epsilon(q)\left[1+\epsilon(q)+(1-\epsilon(q))e^{-\beta}\right]\left[\beta-1+e^{-\beta}\right]}{\beta\left[1+\epsilon(q)+(1-\epsilon(q))e^{-\beta}\right]+2\left(e^{-\beta}-1\right)}, \qquad \beta=qd, \tag{S69}$$

where d is the effective layer thickness. The quantity $\epsilon(q)$ is taken from the bulk dielectric model of Cappellini *et al.* [34],

$$\epsilon(q)=1+\frac{1}{[\kappa-1]^{-1}+\alpha(q/q_{TF})^2+\left(\hbar q^2/(2m\omega_p)\right)^2}, \tag{S70}$$

where q_{TF} is the Thomas-Fermi wavevector, κ is the long-wavelength limit of the static bulk dielectric function, ω_p is the plasma frequency and $\alpha=1.5$. This model is used only for the equilibrium, static, macroscopic screening background $W_{00}(\mathbf{q},0)$. The nonequilibrium evolution is then incorporated through the time-dependent correction $\Delta\epsilon^{-1}_{GG'}(\mathbf{q},t)$ of Eqs. (S38)–(S40), which describes the screening change induced by the evolving photoexcited electron–hole plasma. In this way, the screened Coulomb interaction is updated during the propagation to account for transient renormalization effects associated with the instantaneous carrier distribution.

The resulting scheme combines a model description of the equilibrium macroscopic screening with a first-principles treatment of its nonequilibrium variation. In particular, $\Delta\epsilon^{-1}_{GG'}(\mathbf{q},t)$ is evaluated from first-principles quantities, namely the electronic structure, oscillator strengths, and instantaneous carrier populations, whereas the equilibrium $G=G'=0$ component is described by the isotropic effective 2D model of Ref. [33]. This approach provides a realistic and computationally efficient ground-state baseline: as shown in Ref. [33], the model reproduces the correct strict-2D limit [35] and is in good agreement with experiments and reference *ab initio* calculations for representative layered materials.

10 Brillouin-zone sampling and broadening

In this work, the Brillouin-zone sampling and the numerical broadening are chosen to ensure a reliable description of the quantities that directly control the nonequilibrium dynamics, while keeping the calculations computationally tractable. In practice, the relevant notion of convergence depends on the observable of interest. For the real-time simulations in MoS_2, the key quantities are the collision integrals entering the coupled electron and phonon equations of motion, since they determine the instantaneous scattering strength and therefore the characteristic relaxation timescales. For hBN, instead, the quantities of interest are quasi-stationary, namely the optical absorption and the photoinduced phonon renormalization.

For MoS_2, we assess the sensitivity to $\mathbf{k}/\mathbf{q}$ sampling and smearing by propagating the coupled equations of motion on different momentum grids and with different broadenings, and by monitoring representative measures of the scattering strength at $t=15$ fs, i.e., corresponding to the maximum of the pump pulse. As compact indicators, we consider the Brillouin-zone-averaged magnitudes of the collision integrals,

$$R_X^{\mathrm{el}}=\frac{1}{N}\sum_{\mathbf{k},n}\left|\mathcal{I}^X_{\mathbf{k}n}\right|, \qquad R_X^{\mathrm{ph}}=\frac{1}{N}\sum_{\mathbf{q},\nu}\left|\mathcal{I}^X_{\mathbf{q}\nu}\right|, \tag{S71}$$

where $X=ee$, ep, pe, pp labels the carrier–carrier, carrier–phonon, phonon–electron, and phonon–phonon channels. These averages provide a direct measure of how strongly each interaction channel redistributes electronic or phononic occupations at a given stage of the dynamics.

The convergence trends for these quantities are reported in Fig. S1. As expected, increasing the mesh density and reducing the smearing lead to progressively more stable values of the averaged collision terms. The $36\times36\times1$ grid adopted in the manuscript differs from the $48\times48\times1$ reference only by a few percent for the carrier–phonon and phonon–phonon channels. The carrier–carrier contribution is more demanding with respect to Brillouin-zone sampling, and at the working smearing the difference between the $36\times36\times1$ and $48\times48\times1$ grids is about 15%. Achieving a fully converged description of this channel

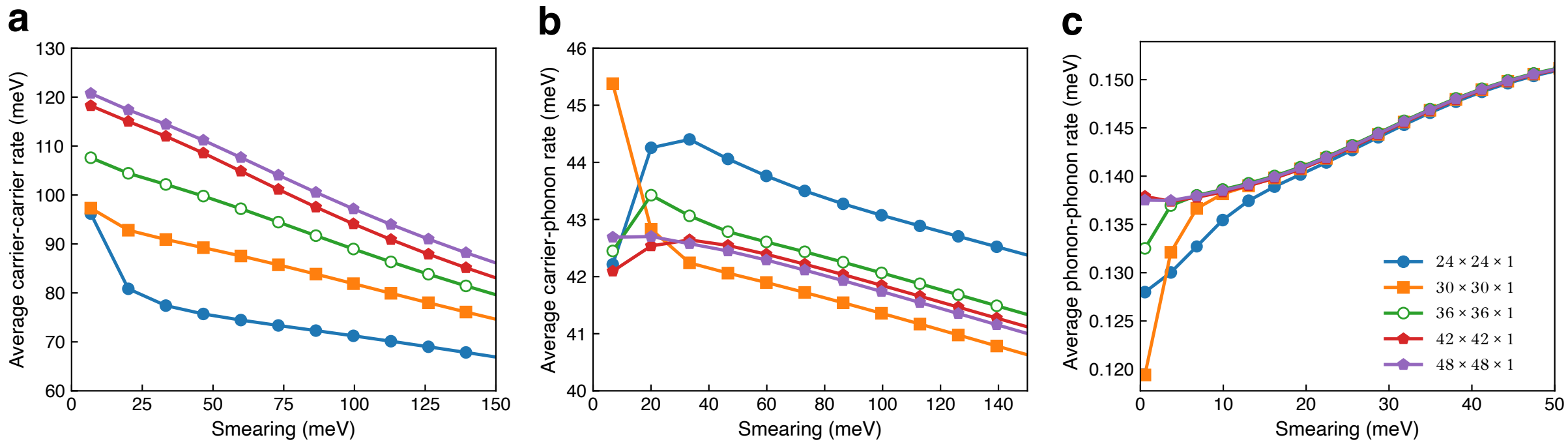

Figure S1: Convergence study of the Brillouin-zone-averaged collision integrals in photoexcited MoS_2 at $t = 15$ fs. Five momentum grids are shown as functions of the numerical broadening. (a) Average electronic collision integral due to carrier–carrier scattering. (b) Average electronic collision integral due to carrier–phonon scattering. (c) Average phonon collision integral due to phonon–phonon scattering.

would require denser meshes, such as $42 \times 42 \times 1$ or $48 \times 48 \times 1$, with a substantially higher computational cost.

Based on these trends, the $36 \times 36 \times 1$ grid represents a practical compromise between numerical accuracy and computational efficiency for the full nonequilibrium propagation in MoS_2. The residual uncertainty mainly affects the carrier–carrier scattering strength and therefore leads to moderate quantitative variations in the corresponding relaxation timescale. By contrast, the relative hierarchy of scattering channels and the overall energy-flow pathways remain unchanged. The sampling used in the manuscript is therefore sufficient for a realistic description of the physical mechanisms discussed in the main text.

For hBN, as representative observables, we consider: (i) the absorption spectrum at vanishing photocarrier density, and (ii) the phonon-frequency renormalization induced by a photocarrier concentration of 5×10^{13} e^-/cm^2. The calculations are carried out on several momentum grids, namely $24 \times 24 \times 1$, $36 \times 36 \times 1$, $48 \times 48 \times 1$, and $60 \times 60 \times 1$.

To quantify the convergence of the phonon renormalization, we introduce the Brillouin-zone-averaged frequency shift

$$\Delta\omega = \frac{1}{N} \sum_{\mathbf{q},\nu} \left| \omega_{\mathbf{q}\nu} - \omega_{\mathbf{q}\nu}^{\text{ref}} \right| , \tag{S72}$$

where $\omega_{\mathbf{q}\nu}^{\text{ref}}$ is the reference result obtained on a $128 \times 128 \times 1$ grid. The corresponding convergence behavior is shown in Fig. S2. Both the absorption spectrum and the averaged phonon-frequency shift display a clear saturation with increasing mesh density. In particular, the variations become marginal beyond $60 \times 60 \times 1$, and the $64 \times 64 \times 1$ grid used in the manuscript, together with the chosen smearing, lies well within the converged regime.

This analysis shows that the numerical parameters adopted for hBN are not only stable with respect to further grid refinement, but also appropriate for a quantitatively reliable description of the photoinduced electronic, optical, and lattice effects discussed in the main text.

11 Relation between electric field and laser fluence

We assume that the incoming electric field is linearly polarized in the xy plane, uniform and non-zero within a planar region of area S. Its time dependence is described by

$$\mathbf{E}(t) = \mathbf{E}_0 \exp\left[-\frac{(t-t_0)^2}{2\Delta t^2} \right] \sin(\omega t), \tag{S73}$$

where $\mathbf{E}_0 = E_0 \boldsymbol{\xi}$, with $\boldsymbol{\xi}$ a unit vector lying in the xy plane.

The instantaneous energy density $u_e(t)$ stored in the electric field is

$$u_e(t) = \frac{1}{2} \epsilon_0 [E(t)]^2. \tag{S74}$$

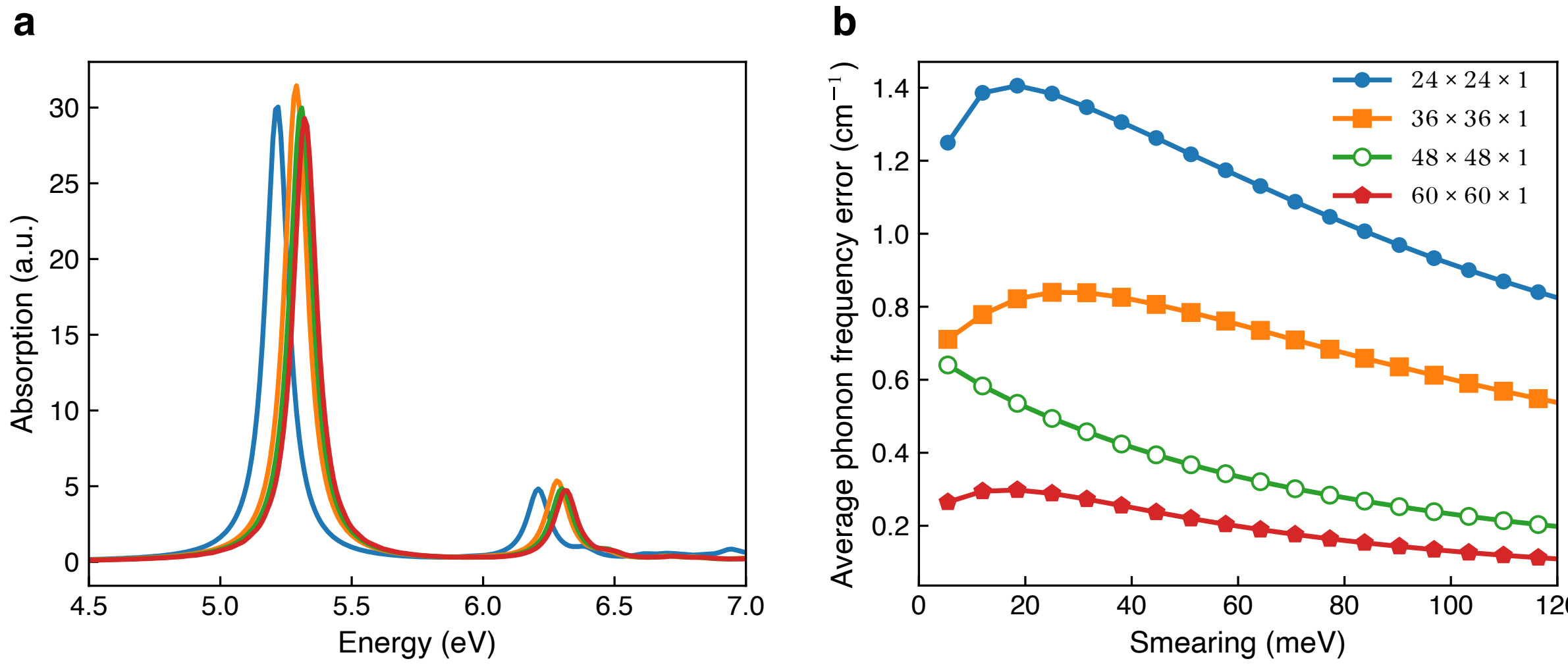

Figure S2: Convergence of quasi-equilibrium hBN observables as a function of the $\mathbf{k}/\mathbf{q}$-grid density: (i) absorption spectrum at vanishing photocarrier density and (ii) Brillouin-zone-averaged phonon-frequency renormalization $\Delta\omega$ at a photocarrier concentration of 5×10^{13} e$^-$/cm^2. The reference phonon renormalization is computed on a $128 \times 128 \times 1$ grid.

From this expression, the infinitesimal energy $dU(t)$ transmitted during a time interval dt through an infinitesimal surface element dS reads

$$dU(t) = \frac{1}{2} c\epsilon_0 [E(t)]^2 \, dS \, dt. \tag{S75}$$

Assuming that the electric field is homogeneous within the region of area S, the laser fluence, defined as the total transmitted energy per unit area, is obtained by integrating over time:

$$\begin{aligned} F = \frac{U}{S} &= \frac{1}{2} c\epsilon_0 \int_{-\infty}^{+\infty} [E(t)]^2 \, dt \\ &= \frac{1}{2} c\epsilon_0 \Delta t E_0^2 \int_{-\infty}^{+\infty} e^{-x^2} \sin^2(\omega \Delta t x - \omega t_0) \, dx \\ &= \frac{\sqrt{\pi}}{4} c\epsilon_0 \Delta t E_0^2 \left[1 - e^{-(\omega\Delta t)^2} \cos(2\omega t_0) \right]. \end{aligned} \tag{S76}$$

For typical values of ω and Δt, the condition $\omega\Delta t \gg 1$ holds, leading to the simplified expression

$$F \simeq \frac{\sqrt{\pi}}{4} c\epsilon_0 \Delta t E_0^2. \tag{S77}$$

References

[1] Konstantinov, O. & Perel, V. A diagram technique for evaluating transport quantities. *SOVIET PHYSICS JETP-USSR* **12**, 142–149 (1961).

[2] Stefanucci, G., van Leeuwen, R. & Perfetto, E. In and out-of-equilibrium ab initio theory of electrons and phonons. *Phys. Rev. X* **13**, 031026 (2023).

[3] Danielewicz, P. Quantum theory of nonequilibrium processes, i. *Annals of Physics* **152**, 239–304 (1984).

[4] Kadanoff, L. P. & Baym, G. *Quantum Statistical Mechanics: Green's Function Methods in Equilibrium and Nonequilibrium Problems* (CRC Press, 2018).

[5] Strinati, G. Application of the green's functions method to the study of the optical properties of semiconductors. *La Rivista del Nuovo Cimento (1978-1999)* **11**, 1–86 (1988).

[6] Maciejko, J. An introduction to nonequilibrium many-body theory. *Lecture Notes, Springer* **9**, 104 (2007).

[7] Lipavský, P., Špička, V. & Velický, B. Generalized kadanoff-baym ansatz for deriving quantum transport equations. *Phys. Rev. B* **34**, 6933–6942 (1986).

[8] Schäfer, W. & Wegener, M. *Semiconductor optics and transport phenomena* (Springer, 2002).

[9] Haug, H. & Jauho, A.-P. *Quantum Kinetics in transport and optics of semiconductors Hartmut haug; Antti-Pekka Jauho* (Springer, 2010).

[10] Stefanucci, G. & Perfetto, E. Semiconductor electron-phonon equations: A rung above Boltzmann in the many-body ladder. *SciPost Phys.* **16**, 073 (2024).

[11] Karlsson, D., van Leeuwen, R., Pavlyukh, Y., Perfetto, E. & Stefanucci, G. Fast green's function method for ultrafast electron-boson dynamics. *Phys. Rev. Lett.* **127**, 036402 (2021).

[12] Marini, A. Competition between the electronic and phonon–mediated scattering channels in the out–of–equilibrium carrier dynamics of semiconductors: an ab-initio approach. *Journal of Physics: Conference Series* **427**, 012003 (2013).

[13] Marini, A., Poncé, S. & Gonze, X. Many-body perturbation theory approach to the electron-phonon interaction with density-functional theory as a starting point. *Phys. Rev. B* **91**, 224310 (2015).

[14] Lafuente-Bartolome, J. *et al.* Ab initio self-consistent many-body theory of polarons at all couplings. *Phys. Rev. B* **106**, 075119 (2022).

[15] Hedin, L. New method for calculating the one-particle green's function with application to the electron-gas problem. *Phys. Rev.* **139**, A796–A823 (1965).

[16] Steinhoff, A. *et al.* Nonequilibrium carrier dynamics in transition metal dichalcogenide semiconductors. *2D Materials* **3**, 031006 (2016).

[17] Steinhoff, A. *et al.* Exciton fission in monolayer transition metal dichalcogenide semiconductors. *Nature Communications* **8** (2017).

[18] Erben, D. *et al.* Excitation-induced transition to indirect band gaps in atomically thin transition-metal dichalcogenide semiconductors. *Phys. Rev. B* **98**, 035434 (2018).

[19] Allen, P. B. & Heine, V. Theory of the temperature dependence of electronic band structures. *Journal of Physics C: Solid State Physics* **9**, 2305 (1976).

[20] Giustino, F. Electron-phonon interactions from first principles. *Rev. Mod. Phys.* **89**, 015003 (2017).

[21] Attaccalite, C., Grüning, M. & Marini, A. Real-time approach to the optical properties of solids and nanostructures: Time-dependent bethe-salpeter equation. *Phys. Rev. B* **84**, 245110 (2011).

[22] Calandra, M., Profeta, G. & Mauri, F. Adiabatic and nonadiabatic phonon dispersion in a wannier function approach. *Phys. Rev. B* **82**, 165111 (2010).

[23] Berges, J., Girotto, N., Wehling, T., Marzari, N. & Poncé, S. Phonon self-energy corrections: To screen, or not to screen. *Phys. Rev. X* **13**, 041009 (2023).

[24] Caldarelli, G., Guandalini, A., Macheda, F. & Mauri, F. Variational formulation of dynamical electronic response functions in the presence of nonlocal exchange interactions. *Phys. Rev. B* **111**, 075137 (2025).

[25] Maradudin, A. A. & Fein, A. E. Scattering of neutrons by an anharmonic crystal. *Phys. Rev.* **128**, 2589–2608 (1962).

[26] Paulatto, L., Errea, I., Calandra, M. & Mauri, F. First-principles calculations of phonon frequencies, lifetimes, and spectral functions from weak to strong anharmonicity: The example of palladium hydrides. *Phys. Rev. B* **91**, 054304 (2015).

[27] Monacelli, L. *et al.* The stochastic self-consistent harmonic approximation: calculating vibrational properties of materials with full quantum and anharmonic effects. *Journal of Physics: Condensed Matter* **33**, 363001 (2021).

[28] Aryasetiawan, F. *et al.* Frequency-dependent local interactions and low-energy effective models from electronic structure calculations. *Phys. Rev. B* **70**, 195104 (2004).

[29] Allen, P. B. Neutron spectroscopy of superconductors. *Phys. Rev. B* **6**, 2577–2579 (1972).

[30] O'Mahony, S. M. *et al.* Ultrafast relaxation of symmetry-breaking photo-induced atomic forces. *Phys. Rev. Lett.* **123**, 087401 (2019).

[31] Janak, J. F. Proof that $\frac{\partial e}{\partial n_i} = \epsilon$ in density-functional theory. *Phys. Rev. B* **18**, 7165–7168 (1978).

[32] Dormand, J. & Prince, P. A family of embedded runge-kutta formulae. *Journal of Computational and Applied Mathematics* **6**, 19–26 (1980).

[33] Trolle, M. L., Pedersen, T. G. & Véniard, V. Model dielectric function for 2d semiconductors including substrate screening. *Scientific Reports* **7**, 39844 (2017).

[34] Cappellini, G., Del Sole, R., Reining, L. & Bechstedt, F. Model dielectric function for semiconductors. *Phys. Rev. B* **47**, 9892–9895 (1993). URL https://link.aps.org/doi/10.1103/PhysRevB.47.9892.

[35] Cudazzo, P., Tokatly, I. V. & Rubio, A. Dielectric screening in two-dimensional insulators: Implications for excitonic and impurity states in graphane. *Phys. Rev. B* **84**, 085406 (2011). URL https://link.aps.org/doi/10.1103/PhysRevB.84.085406.
